# How digital health tools shape emotional support experiences in cardiovascular care: a systematic review and thematic synthesis

**DOI:** 10.3389/fcvm.2026.1923477

**Published:** 2026-08-13

**Authors:** Junru Zeng, Hong Wang

**Affiliations:** School of Physical Education, Wuhan Sports University, Wuhan, China

**Keywords:** cardiovascular disease, digital health, emotional support, mobile health, patient experience, qualitative evidence synthesis, telemedicine, trust

## Abstract

**Background:**

Digital health tools are increasingly integrated into cardiovascular care, yet their value is commonly assessed through clinical outcomes, adherence, and usability. Less is known about how patients experience these tools as sources of emotional support, or how digitally mediated care can instead create emotional burden. In this review, emotional support refers to patients’ experiences of feeling reassured, understood, encouraged, validated, and relationally connected while managing cardiovascular illness.

**Objective:**

To synthesize qualitative evidence on how digital health tools shape emotional support experiences among patients with cardiovascular disease or closely related cardiovascular conditions, and to identify the mechanisms and contextual factors that determine whether technology is experienced as supportive or burdensome.

**Methods:**

This systematic review and qualitative synthesis was designed in accordance with the PRISMA 2020 and ENTREQ guidelines. PubMed, Web of Science, Embase, Scopus, CNKI, Wanfang, and VIP databases were searched from inception to December 2025. Studies reporting qualitative patient data on mobile applications, remote monitoring, telerehabilitation, conversational artificial intelligence, virtual reality, or related digital tools in cardiovascular care were included. Methodological quality was appraised using the Joanna Briggs Institute (JBI) checklist. Findings were integrated through thematic synthesis, and confidence in the evidence was assessed using GRADE-CERQual.

**Results:**

Eighteen studies, representing approximately 401 patients across 14 countries or regions, were included. Five analytical themes were generated: (1) reassurance through monitored and responsive care; (2) agency through understandable and actionable information; (3) relational continuity through humanized digital connection; (4) motivation and hope through personalization, enjoyment, and encouragement; and (5) distress and disengagement through technical, informational, and ethical burden. Six cross-cutting contextual moderators shaped these pathways: human-in-the-loop support, technical reliability, personalization, cultural safety, digital and health literacy, and continuity with routine care.

**Conclusion:**

Emotional support is not an inherent property of a digital tool. It is an experience co-produced when technology enables credible oversight, understandable feedback, responsive relationships, and patient control. Cardiovascular digital interventions should therefore be evaluated not only for usability and clinical effectiveness, but also for reassurance, relational continuity, anxiety, trust, and digital treatment burden.

**Systematic Review Registration:**

CRD420261430511.

## 1 Introduction

Cardiovascular disease imposes sustained demands for symptom appraisal, medication use, lifestyle change, and decisions about when to seek care. These demands often occur alongside uncertainty, fear of deterioration, anxiety about bodily sensations, and concerns about safely resuming everyday activities (1). Supportive interpersonal communication can reduce uncertainty and help patients cope, whereas low perceived support is associated with poorer cardiovascular adjustment and outcomes (2).

Digital health tools—including mobile applications, remote patient monitoring, web-based cardiac rehabilitation, conversational artificial intelligence (AI), and immersive virtual reality—are increasingly used to extend cardiovascular care beyond hospitals and clinics. They can provide monitoring, feedback, education, behavioral prompts, and access to clinicians (3, 4, 12). However, technical functionality does not determine emotional experience. The same measurement may reassure a patient when a clinician is visibly reviewing it, but provoke hypervigilance when its meaning and response pathway are unclear. Likewise, an automated message may be encouraging when relevant and personalized, yet feel intrusive or impersonal when repetitive or poorly timed.

Existing reviews of digital cardiovascular care have mainly synthesized adoption, usability, engagement, and barriers and facilitators (5), while a recent qualitative meta-synthesis identified emotional and relational value as one component of the wider patient experience (6). The present review differs by treating emotional support as the primary phenomenon of interest and by examining the mechanisms and contextual conditions through which digital care becomes emotionally supportive or burdensome.

For this review, emotional support was conceptually anchored in the emotional-support dimension of social support: communication or relational action that conveys care, empathy, reassurance, encouragement, validation, and a sense that help is available (45, 46). The core phenomenon was therefore the patient's experienced sense of reassurance, being understood, encouragement, validation, or relational connection. Related concepts were not treated as interchangeable synonyms. Trust, perceived control/agency, and connectedness were coded as mechanisms or proximal conditions that could enable emotional support; anxiety, fear, frustration, treatment burden, and disengagement were coded as negative emotional outcomes or boundary conditions indicating that support had failed. This distinction guided eligibility, coding, and interpretation. The review asked: (1) How do patients describe emotionally supportive or burdensome experiences associated with digital cardiovascular care? (2) Through what technical and interpersonal mechanisms do these experiences arise? and (3) Which contextual conditions modify these pathways?

## 2 Methods

### 2.1 Design and reporting

A systematic review with thematic synthesis was undertaken. We use “thematic synthesis” rather than the broader label “meta-synthesis” because the analysis followed the specific three-stage method described by Thomas and Harden: line-by-line coding, development of descriptive themes, and generation of analytical themes (10). Reporting followed PRISMA 2020 and ENTREQ (7, 8). The protocol was registered in PROSPERO (CRD420261430511). Completed PRISMA 2020 and ENTREQ checklists are provided in the Supplementary Material.

### 2.2 Inclusion criteria

Eligibility was structured using the Population–Interest–Context (PICo) framework (Table 1). The population comprised adults with established cardiovascular disease or a clinically significant cardiovascular condition, including heart failure, coronary heart disease, myocardial infarction or percutaneous coronary intervention, atrial fibrillation, hypertension, and hypertensive disorders of pregnancy. Cardiometabolic-risk populations were eligible only when the digital intervention and qualitative findings directly concerned cardiovascular self-management.

**Table 1:** Population–interest–context eligibility criteria.

PICo element	Inclusion criteria	Exclusion criteria	Role in emotional-support framework
Population	Adults with cardiovascular disease or clinically significant cardiovascular conditions; cardiometabolic-risk samples only when findings directly concerned cardiovascular self-management.	Non-cardiovascular populations; pediatric-only samples; clinician-only data without extractable patient findings.	Defines whose first-person experience was synthesized.
Interest	Core experiences of reassurance, being understood, encouragement, validation, or relational connection; mechanisms (trust, agency/control, connectedness); negative outcomes/boundaries (anxiety, fear, frustration, burden, privacy concern, distrust, disengagement) explicitly linked to digital care.	Studies reporting usability or adherence without an emotional/relational link; quantitative outcomes without qualitative patient accounts.	Separates the core phenomenon from mechanisms and negative boundary conditions.
Context	Mobile apps, remote monitoring, telehealth/telerehabilitation, conversational AI, virtual reality, or related digital tools in home, community, outpatient, post-discharge, rehabilitation, or blended care.	Non-digital interventions; purely inpatient technology without a patient-experience component.	Captures the sociotechnical setting in which support is mediated.
Study type/language	Original qualitative studies and mixed-methods studies with extractable qualitative data; English or Chinese.	Quantitative-only studies, protocols, reviews, editorials, conference abstracts without sufficient qualitative findings.	Ensures data are sufficiently rich for thematic synthesis.

The interest was patients' first-person experience of emotional support or emotional burden during actual use, consideration, or discontinuation of a digital health tool. Eligible findings had to contain the core experience (reassurance, feeling understood, encouragement, validation, or relational connection) or evidence about a prespecified mechanism/boundary condition directly linked to that experience. Trust, agency/perceived control, and connectedness were treated as enabling mechanisms; anxiety, fear, distress, frustration, privacy concern, treatment burden, and disengagement were treated as negative outcomes or boundary conditions. These sensitizing concepts were prespecified in the protocol and search strategy, while the final descriptive categories and analytical themes were developed inductively from the included findings.

Contexts included home, community, outpatient, post-discharge, rehabilitation, and blended-care settings. Original qualitative studies and mixed-methods studies with extractable qualitative patient data were included. Quantitative-only studies, protocols, reviews, editorials, non-cardiovascular populations, studies without patient perspectives, and studies without findings relevant to the defined phenomenon were excluded. Studies in English or Chinese were eligible.

### 2.3 Information sources and search strategy

PubMed, Embase, Web of Science, Scopus, CNKI, Wanfang, and VIP were searched from inception to December 2025. Search terms covered cardiovascular conditions, digital technologies, emotional or relational experience, and qualitative methods. Database-specific strategies and the number of records retrieved from each database are reported in Supplementary Appendix S1. Reference lists of included studies and relevant reviews were backward searched to identify additional eligible reports. Grey literature was not searched because the synthesis required sufficiently detailed, peer-reviewed qualitative findings for line-by-line analysis; this decision is acknowledged as a limitation.

### 2.4 Study selection

Study screening was conducted independently by two reviewers (S.F.R. and Z.J.R.) in two stages. Titles and abstracts were assessed for eligibility in the initial screening phase, followed by full-text review of potentially eligible articles.

### 2.5 Data extraction

A standardized form captured author, year, country or region, cardiovascular condition, sample, digital technology, intervention or use context, duration, study design, data collection and analysis methods, the person or service providing human support behind the technology, and all relevant first- and second-order findings. Results text, authors' thematic interpretations, and participant quotations were treated as qualitative data. Distinguishing the tool from the human support provider allowed us to examine whether support was attributed to technology itself or to staff, peers, or family members connected through it.

### 2.6 Methodological quality appraisal

Methodological quality was appraised using the Joanna Briggs Institute Critical Appraisal Checklist for Qualitative Research (10 items) (9). Appraisal considered congruence between methodology, research question, data collection, analysis, and interpretation; researcher positionality and reflexivity; representativeness of participants; ethical conduct; and whether conclusions were derived from the data. Quality was used to guide interpretation and confidence rather than as an automatic exclusion threshold.

### 2.7 Data synthesis

Thematic synthesis followed Thomas and Harden's three stages (10). First, two reviewers independently coded eligible findings line by line. Coding combined deductive and inductive elements. A small set of protocol-defined sensitizing concepts—core emotional-support experiences, enabling mechanisms, and negative boundary conditions—ensured conceptual consistency, while new codes were added when findings did not fit the initial framework. Second, reviewers compared codes across studies and grouped them inductively into descriptive categories that remained close to the primary data (for example, “being watched over,” “understanding what the numbers mean,” “access to a real person,” and “technology becoming another task”). These categories were not predefined. Third, the team developed analytical themes that moved beyond individual studies to explain how digital features, human actors, and contexts interacted to produce supportive or burdensome experiences. Disagreements were resolved through discussion, and negative cases were retained to test the boundaries of each interpretation.

The “emotional-support lens” therefore did not mean that every positive or negative user experience was automatically classified as emotional support. Coding required an explicit link to the core experience or to a mechanism/boundary condition affecting it. For example, remote monitoring was coded as a technological feature; perceived professional oversight and interpretive feedback were coded as mechanisms; reassurance was coded as the supportive experience; and monitoring anxiety or distrust was coded as a negative outcome. This analytic separation enabled comparison across heterogeneous tools without collapsing usability, empowerment, trust, and emotion into a single construct.

### 2.8 Confidence in synthesized findings

GRADE-CERQual was used to assess the confidence of each analytical finding, considering methodological limitations, coherence, adequacy, and relevance (11). Confidence judgments were categorized as high, moderate, low, or very low.

## 3 Results

### 3.1 Study selection

**Figure 1:**
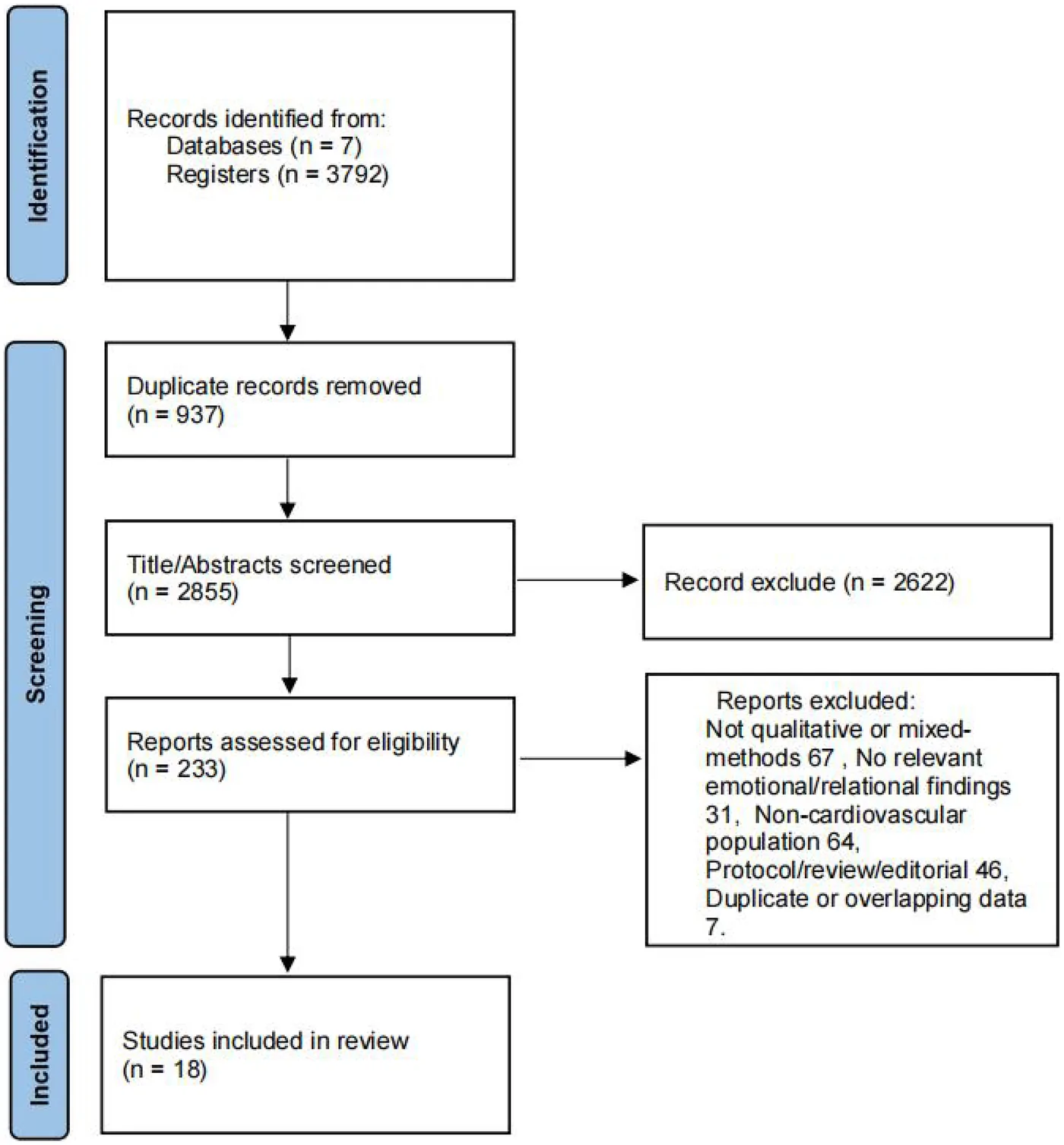
Flow diagram.

The searches retrieved 3,792 records: PubMed (458), Embase (532), Web of Science (1,491), Scopus (1,115), CNKI (86), Wanfang (67), and VIP (43). After duplicate removal, 2,855 records underwent title and abstract screening; 233 full texts were assessed, and 18 studies were included. Backward reference checking did not identify additional eligible reports. The selection process is shown in Figure 1.

### 3.2 Study characteristics

The 18 studies represented approximately 401 patient participants or interview records across 14 countries or regions: Australia, Belgium, Canada, mainland China, Germany, Hong Kong SAR, Ireland, Italy, Macao SAR, the Netherlands, Singapore, South Korea, Sweden, the United Kingdom, and the United States (some studies were multicountry). Populations included heart failure, coronary heart disease, post-myocardial infarction or post-PCI, atrial fibrillation, hypertension, hypertensive disorders of pregnancy, and one cardiometabolic high-risk community sample. Tools included mobile self-management applications, eHealth and telerehabilitation platforms, remote blood-pressure monitoring, conversational AI, immersive virtual reality, and generic digital products. Human support, when present, was provided by nurses, pharmacists, physiotherapists, cardiologists or other clinicians, peers, and family members. Three studies explored anticipated use; the remainder examined actual use. Table 2 summarizes the study and support-provider characteristics.

**Table 2:** Characteristics of included studies and human support connected to the digital tool.

Study	Country/region	Population (n)	Digital tool	Design/exposure	Human support connected to tool	Key emotional-support finding
O'Shea et al. (2020) (29)	Ireland/Belgium	CVD patients after cardiac rehabilitation	PATHway phase III eHealth cardiac rehabilitation system	Qualitative interviews after a 6-month intervention	Physiotherapists/rehabilitation team	Reassurance about safe exercise; motivation and accountability; frustration with complex equipment and variable engagement.
Foreman et al. (2025) (26)	United States	Black pregnant people with hypertensive disorders of pregnancy	Digital health tools used or considered during pregnancy	Grounded-theory interviews	Clinicians, family, and social support networks	Support systems, autonomy, and provider trust facilitated engagement; discrimination and mistrust undermined emotional safety.
Choo et al. (2025) (44)	South Korea	Community adults with metabolic-syndrome criteria	My HeartHELP mobile app	Mixed-methods feasibility study with focus groups; 4 weeks	Automated tailored feedback; research team	Automated tailored feedback encouraged self-monitoring and motivation; users requested more sympathetic, individualized feedback.
Su et al. (2023)	Hong Kong, China	Patients with coronary heart disease	Nurse-led eHealth cardiac rehabilitation website plus WeChat	Qualitative process evaluation of a 12-week RCT intervention	Nurses and peer participants via WeChat	Reduced post-CHD distress; professional counseling and peer interaction provided reassurance, normalization, and support.
Schmaderer et al. (2021) (19)	United States	Patients recently hospitalized with advanced heart failure	Heart-failure self-management mobile app	Qualitative descriptive interviews	App education; patients’ clinical team	Greater awareness and empowerment; focusing on health felt positive; stronger confidence in communication with the care team.
Blomqvist et al. (2025) (28)	Sweden	People with heart failure	Activity Coach physical-activity app	Thematic interviews after 12 weeks	Primarily app-based; no continuous clinician support	Enjoyment of monitoring, motivation, routine formation, and improved emotional well-being; frustration with tracking limitations.
Chu et al. (2024) (14)	United States	Primary-care patients with uncontrolled hypertension	Cellular home BP monitoring plus nurse/pharmacist calls	Qualitative implementation study	Nurse and pharmacist	Relationship with the team and clinician oversight created security; readings could also trigger stress and distrust.
Trivedi et al. (2025) (17)	Australia	Patients with atrial fibrillation	Voice-based conversational AI plus multichannel education	Qualitative interviews after a 6-month intervention	Automated AI; hospital/clinical service available	Continuity, reminders, and rhythm monitoring were reassuring; restricted dialogue and information volume reduced perceived empathy.
Sivakumar et al. (2023) (47)	Canada	Patients with heart failure	Potential mobile apps for HF management	Qualitative focus groups	Proposed clinicians, peers, and family	Patients wanted clinician, peer, and family connection; credible information offered assurance; privacy and technical failures caused worry.
Neumann et al. (2024) (15)	Germany/European multicountry	Patients with heart failure	DoctorME self-care app	Longitudinal qualitative interviews at 2–3 weeks and 4–6 months	Clinicians expected to interpret data and provide feedback	Continuous monitoring and rapid feedback created security for some; stand-alone use without medical feedback was not acceptable to many.
Madujibeya et al. (2023) (16)	United States	Patients with heart failure in routine care	OnTrack to Health commercial mHealth app	Real-world qualitative survey analysis	Clinic staff via secure messaging/technical support	Assurance of safety, connectedness to the clinic, rapid responses, and motivational alerts; burden from changing established routines.
Son et al. (2020) (24)	South Korea	Patients with chronic heart failure	Prospective mobile-phone intervention concepts	Qualitative interviews before intervention development	Proposed clinicians and family	Patients expected reassurance from easier clinician contact and personalized information; cost, security, and reduced human interaction were concerns.
Nilsson et al. (2023) (13)	Sweden	Patients after myocardial infarction	Exercise-based cardiac telerehabilitation	Inductive qualitative interviews after 3 months	Physiotherapist	Expert physiotherapist supervision reduced fear of movement and created safety; encouragement increased motivation.
Micheluzzi et al. (2025) (27)	Italy	Patients with heart failure in cardiac rehabilitation	Immersive virtual reality during rehabilitation	Qualitative study embedded in a trial	Rehabilitation staff/research team	Positive emotions, distraction, enjoyment, motivation, and anxiety management; headset discomfort and unfamiliarity caused irritation.
Lao and Chair (2022) (20)	Macao, China	Chinese patients after PCI	Smartphone-based cardiac rehabilitation app	Qualitative evaluation embedded in a trial	Nurses/clinical rehabilitation team	Reliable information and nurse communication extended care and strengthened the patient–clinician relationship; usability barriers remained.
Cher et al. (2020) (18)	Singapore	Patients with atrial fibrillation (plus clinicians)	Proposed AF self-management e-tool	Exploratory thematic interviews	Proposed clinicians	Patients prioritized contact with clinicians over autonomous monitoring; overmonitoring was perceived as potentially anxiety-provoking.
Tang et al. (2022) (21)	China	Patients with hypertension	Hospital mobile-health management platform	Phenomenological interviews after ≥6 months of use	Family doctors/clinical team	Timely family-doctor responses created reassurance and reduced illness worry; workload, interface, and privacy concerns affected sustained use.
Lan et al. (2021) (25)	China	Heart-failure patients who discontinued an app	“Heart Butler” post-discharge mHealth app	Qualitative interviews with low-acceptance/discontinuing users	Clinical team during implementation; family assistance	Low usefulness and ease of use produced frustration, burden, inferiority, and negative psychological experiences, contributing to abandonment.

### 3.3 Methodological quality

Methodological quality was appraised using the Joanna Briggs Institute (JBI) Critical Appraisal Checklist for Qualitative Research (10 items), which evaluates congruence between the research methodology, research questions, data collection, analysis, interpretation, researcher reflexivity, ethical considerations, and the representation of participants' voices (9). Consistent with JBI guidance, the purpose of quality appraisal was to inform interpretation of the synthesized findings rather than to exclude studies solely on the basis of methodological limitations (9).

Overall, the included studies were of acceptable to good methodological quality. All 18 studies (100%) clearly stated their research objectives, demonstrated methodological congruence, adequately represented participants' voices, and reported ethical approval and informed consent procedures (Items 1–4 and 8–10).

However, researcher reflexivity was the most frequently underreported domain. Only five studies (28%) adequately described researcher positioning and its potential influence on the research process (Item 5); two studies (11%) explicitly reported the researchers' cultural or theoretical positioning (Item 6); and only one study (6%) described the prior relationship between researchers and participants (Item 7). Previous methodological literature has identified inadequate reporting of researcher reflexivity as a common limitation in qualitative health research, because insufficient reflexivity may affect the transparency and credibility of qualitative interpretations (9, 11).

Blomqvist et al. received adequate ratings across all appraisal items because the investigators explicitly disclosed conflicts of interest and reflected on the potential influence of the researcher's dual role during data interpretation. As recommended in qualitative evidence synthesis methodology, all studies were retained regardless of appraisal score, while methodological limitations were subsequently considered during the GRADE-CERQual assessment of confidence in the review findings (9, 11) (Supplementary Table S2).

### 3.4 Synthesized findings

Five analytical themes explain how digital health tools shape emotional support. The themes are not mutually exclusive; the same feature may produce both support and distress depending on implementation and context.

#### 3.4.1 Reassurance through monitored and responsive care

Across remote monitoring, telerehabilitation, and heart-failure applications, patients described reassurance when data collection was visibly connected to a clinician who could interpret changes and respond. After myocardial infarction, exercise telerehabilitation supervised by a physiotherapist reduced fear of exercise and created a sense of safety (13). In home blood-pressure monitoring, nurse and pharmacist contact conveyed that someone was attending to the patient's condition (14). Heart-failure apps with secure messaging or rapid clinician feedback similarly provided assurance that help remained accessible (15, 16).

Similarities across these settings concerned the response pathway rather than the device: measurements were supportive when patients knew who reviewed them, what abnormal values meant, and what action would follow (13–17). In atrial fibrillation care, rhythm monitoring reassured some patients when results were interpreted within clinical care (17). By contrast, the same monitoring generated stress when readings were ambiguous, device accuracy was doubted, or no response was visible (14, 18). Thus, professional oversight and interpretation, rather than monitoring alone, constituted the mechanism of reassurance.

Technology-specific differences were also evident. Telerehabilitation primarily reduced fear about exercising safely through synchronous or clearly attributable professional supervision (13), whereas home-monitoring apps provided reassurance through asynchronous review, alerts, and messaging (14–16). These formats differed in immediacy, but converged on the need for credible human accountability.

#### 3.4.2 Agency through understandable and actionable information

Across heart-failure apps, hypertension platforms, lifestyle applications, and nurse-led cardiac rehabilitation, understandable information reduced uncertainty and supported a sense of control (19, 21, 23, 24, 44). Heart-failure participants described becoming more aware of symptoms and self-care requirements and more confident in communicating with clinicians (19). Patients also valued having credible, disease-specific information in one place rather than searching fragmented online sources (22, 24).

The common mechanism was translation of data into action. Trend displays, reminders, and tailored feedback were supportive when they helped patients interpret weight, blood pressure, activity, medication, or symptom changes and decide what to do next (15, 16, 21, 23, 44). In My HeartHELP, automated tailored feedback encouraged lifestyle self-monitoring (44); in nurse-led eHealth cardiac rehabilitation, goal setting, counseling, and a structured recovery roadmap reduced post-coronary-disease distress (23).

Differences emerged according to prior knowledge and content volume. Patients new to self-management often valued structured education, whereas some with established routines perceived little added benefit (15). Generic advice, jargon, repetition, or excessive information—especially in conversational AI and proposed apps—could create overload rather than control (17, 22, 24). Information therefore contributed to emotional support only when it was credible, comprehensible, personalized, and proportionate.

#### 3.4.3 Relational continuity through humanized digital connection

Patients consistently valued digital tools as extensions of relationships rather than substitutes for them. In cardiac rehabilitation, hypertension management, and heart-failure care, access to nurses, pharmacists, physiotherapists, cardiologists, or family physicians was described as reassuring (13–16, 20, 21, 23). Patients with atrial fibrillation often prioritized contacting clinicians over independently interpreting medical data (18), while users of the DoctorME app viewed it as a complement to professional guidance (15).

Peer and family support added different relational functions. Peer interaction normalized illness experiences and enabled practical comparison, as reported in heart-failure focus groups and nurse-moderated WeChat cardiac rehabilitation (22, 23). Family members often helped older users operate technology and interpret health information in East Asian settings (20, 21, 24, 25). Unlike clinician support, which supplied interpretive authority and action, peer and family support primarily supplied experiential understanding, encouragement, and practical assistance.

Conversational AI marked the boundary of digital substitution. Voice calls could feel engaging and provide continuity and reminders, but restricted response options and scripted dialogue limited natural conversation and perceived empathy (17). The finding was not that AI could never support patients, but that emotionally complex concerns still required access to a responsive person.

Cultural safety further conditioned relational continuity. Black pregnant participants with hypertensive disorders described supportive networks, bodily autonomy, and provider trust as facilitators, while discriminatory encounters undermined emotional safety and expectations of digital care (26). Cultural tailoring alone could not compensate for unresponsive or inequitable clinical relationships.

#### 3.4.4 Motivation and hope through personalization, enjoyment, and encouragement

Positive affect was most prominent in immersive virtual reality, physical-activity apps, and rehabilitation programs. Immersive virtual reality made repetitive rehabilitation more engaging, altered time perception, and helped manage anxiety, although headset discomfort could produce irritation (27). A heart-failure activity app supported enjoyment, awareness, routine formation, and perceived emotional improvement, despite limitations in activity tracking (28).

Across PATHway, My HeartHELP, and telerehabilitation, tailored prompts, visible progress, and encouraging professional feedback supported motivation (13, 29, 44). The source of encouragement differed: automated messages were valued when personally relevant, while physiotherapist encouragement carried relational credibility. Across both formats, hope was sustained when feedback made improvement visible and helped patients establish feasible routines; poorly timed or repetitive prompts could instead feel controlling (13, 28, 29, 44).

#### 3.4.5 Distress and disengagement through technical, informational, and ethical burden

Technical complexity, device incompatibility, unreliable connectivity, cumbersome data entry, poor interface design, and hardware discomfort caused frustration across mobile apps, multicomponent eHealth systems, remote monitoring, and immersive virtual reality (14, 22, 25, 27–29). Older adults and patients with visual, cognitive, educational, or functional limitations described dependence, embarrassment, or reduced confidence when operation required multiple steps or repeated troubleshooting (22, 24, 25).

Ethical and informational burdens also undermined trust. Privacy and data-sharing concerns appeared in heart-failure, hypertension, pregnancy, and proposed-app studies (21, 22, 24, 26). Cost, excessive messages, uncertainty about device accuracy, and fear that technology would reduce human contact added to the workload of illness (14, 17, 22, 24). The discontinuation study provided a particularly clear negative case: low perceived usefulness and ease of use were accompanied by frustration, inferiority, and abandonment (25).

Across tools, the similarity was a reversal pathway: when the effort, uncertainty, or perceived risk introduced by technology exceeded the support gained, digital care became an additional treatment burden. The specific burden varied by tool—equipment complexity in PATHway, tracking limitations in activity apps, discomfort in virtual reality, or privacy and accuracy concerns in monitoring—but the emotional consequence converged on anxiety, distrust, or disengagement (14, 25, 27–29).

### 3.5 Cross-cutting contextual moderators and confidence in findings

The analytical themes describe recurring patterns of patient experience, whereas the six cross-cutting moderators describe conditions that altered whether those patterns became supportive or burdensome. (1) Human-in-the-loop support concerned whether a recognizable person reviewed information and could respond. (2) Personalization concerned fit with disease stage, symptoms, knowledge, goals, and communication preferences. (3) Technical reliability concerned accuracy, connectivity, interoperability, and low-friction operation. (4) Digital and health literacy support concerned onboarding, accessible language, and help with interpretation. (5) Cultural safety concerned respect, autonomy, representation, and freedom from discriminatory care. (6) Care continuity concerned integration with routine services and clarity about responsibility. These moderators were generated by comparing conditions repeatedly present in positive and negative cases across all five themes; they are contextual explanations, not additional themes. Table 3 summarizes the CERQual judgments.

**Table 3:** CERQual summary of synthesized findings.

Analytical theme	Contributing evidence	Main concerns	CERQual confidence	Interpretation
Professional oversight converts monitoring into reassurance	Consistent across telerehabilitation, RPM, HF apps, AF monitoring, and cardiac rehabilitation	Some self-selection; variable intervention designs	High	Robust and coherent across conditions and technologies.
Information and feedback restore agency	Broad evidence from HF, hypertension, CHD, AF, and lifestyle apps	Some studies assessed anticipated rather than actual use	High	Strong convergence, with clear negative cases involving overload.
Human relationships mediate digital emotional support	Clinician, peer, and family connection reported across regions	Peer-support evidence less extensive than clinician-support evidence	Moderate to high	Highly coherent, but relational models varied.
Enjoyment and encouragement sustain motivation	Strong in VR, physical-activity apps, rehabilitation, and tailored feedback	Fewer studies and mostly short intervention periods	Moderate	Plausible and coherent but long-term durability is uncertain.
Technical and ethical burden can produce distress and disengagement	Reported in nearly all technology categories, including discontinuers	Severity and frequency not quantifiable from qualitative data	High	Negative effects were consistent and supported by explicit accounts.

## 4 Discussion

### 4.1 Principal findings

This thematic synthesis of 18 studies shows that digital cardiovascular care becomes emotionally supportive when technical functions are connected to credible interpretation, responsive relationships, and patient control. The five themes represent the principal experiential findings; the six moderators explain why the same feature can produce different outcomes across tools and implementation contexts. Emotional support was not attributed to technology alone: clinicians, peers, and family members frequently supplied the reassurance, validation, interpretation, or encouragement that technology mediated.

### 4.2 Comparison with existing literature

Our findings extend prior reviews in three ways. First, cardiovascular digital-health reviews have largely organized evidence around usability, adoption, engagement, or barriers and facilitators (5, 6), whereas this review differentiates the core experience of emotional support from its mechanisms and negative boundary conditions. Second, our synthesis asks how particular sociotechnical arrangements generate reassurance, connection, encouragement, or burden in patients' own accounts rather than treating emotional outcomes only as secondary measures. Third, the analysis explains a recurring cross-context mechanism: monitoring becomes reassuring only when linked to visible professional oversight and a clear response pathway. This mechanism-oriented contribution complements, rather than duplicates, the broader 2025 meta-synthesis of cardiovascular digital-health experiences (6).

### 4.3 Integrative conceptual model derived from the synthesis

**Figure 2:**
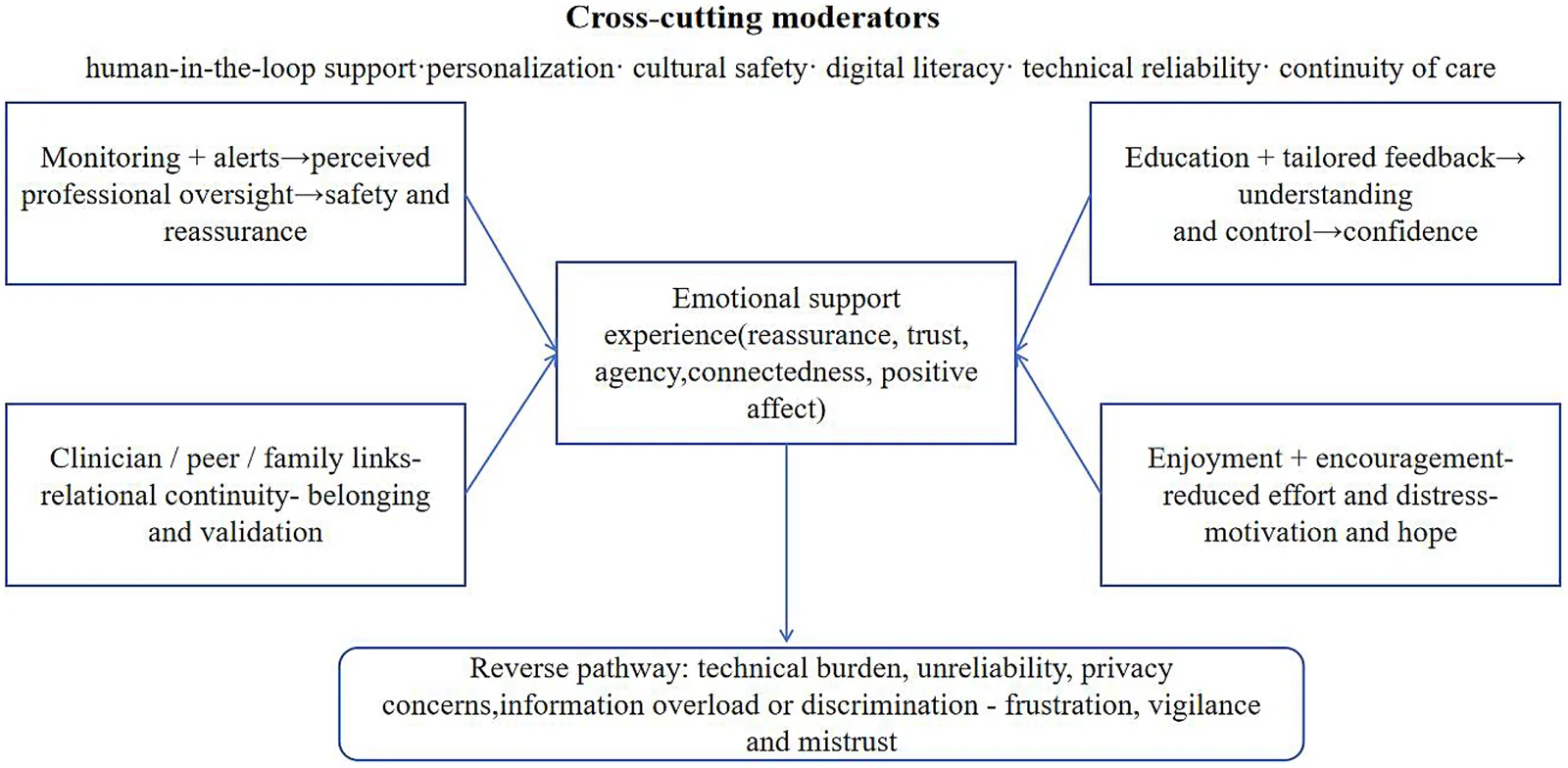
Integrative conceptual model of digitally mediated emotional support in cardiovascular care.

Figure 2 is presented as an integrative conceptual model, or line-of-argument synthesis, derived from the empirical findings—not as a new formal theory. It brings together the five analytical themes and six moderators into a higher-order explanation. Digital features such as monitoring, education, messaging, feedback, or immersive experiences create opportunities for support. These opportunities are translated through mechanisms including perceived oversight, actionable understanding, relational continuity, personalization, and enjoyment. Contextual moderators determine the strength and direction of that translation. Supportive configurations produce reassurance, agency, connection, motivation, and hope; poorly configured systems produce anxiety, distrust, burden, and disengagement. The model therefore explains how similar tools can yield divergent emotional experiences and identifies propositions for future empirical testing.

### 4.4 Information, agency, and the limits of data provision

Our second theme clarifies that information restores emotional equilibrium only when it is actionable, comprehensible, and proportionate to the patient's existing knowledge and contextual needs. While digital education is widely assumed to empower, agency in our synthesis depended on the translation of raw data into meaningful, personalized feedback (22, 30). Generic content, excessive message volume, and medical jargon overwhelmed patients, particularly those with established self-care routines who perceived limited incremental value (31). This finding suggests that emotional support requires curated transparency rather than unrestricted information access, and that the emotional value of data lies in its interpretive framing and actionability rather than in its mere availability. The distinction between data provision and actionable understanding has direct implications for interface design and content architecture in cardiovascular applications.

### 4.5 Relational continuity and the boundaries of digital substitution

The third theme establishes that patients consistently position digital tools as extensions of, rather than substitutes for, human relationships. This challenges techno-optimistic narratives that anticipate autonomous self-management through automation and artificial intelligence. Patients prioritized clinician contact over independent data interpretation (32, 33), and conversational AI despite engaging interface design was perceived as limited in empathy when addressing emotionally complex concerns (34). The value of peer connection further underscores that emotional support involves normalization and experiential understanding that cannot be algorithmically generated (35, 36). These boundaries suggest that digital health implementations should preserve and enhance relational continuity rather than displacing it, particularly in contexts where trust and reassurance depend on perceived clinician accountability.

### 4.6 Cultural safety, equity, and structural trust

The inclusion of marginalized populations (37) highlights a critical dimension often underrepresented in cardiovascular digital health research: cultural safety and structural trust. Digital tools cannot repair distrust arising from discriminatory clinical encounters simply by adding culturally representative content; they must be embedded within respectful, accountable care relationships that acknowledge historical and ongoing inequities. This finding demands that cultural safety be treated as a core design and implementation principle, not an optional feature, particularly for populations bearing disproportionate cardiovascular morbidity. The emotional safety of digital care depends not only on interface design but on the extent to which broader care systems demonstrate respect and responsiveness.

### 4.7 Positive affect, motivation, and the enjoyment paradox

Theme four reveals that positive affect enjoyment, encouragement, and hope plays a distinct role in sustaining engagement that is partially independent of negative-affect reduction. Immersive virtual reality transformed rehabilitation from aversive exertion into engaging experience, altering time perception and supporting anxiety management (38). Similarly, progress visualization and tailored feedback in physical activity applications generated motivation through perceived mastery and routine formation (39, 40). These findings suggest that emotional support in digital health encompasses not only the alleviation of anxiety and fear but also the active generation of positive affect. However, the durability of these effects beyond short intervention periods remains uncertain, warranting longitudinal investigation to determine whether enjoyment translates into sustained behavioral maintenance.

### 4.8 Technical burden and the reversal pathway

Theme five identifies a reversal pathway in which technical and ethical burden transforms potential support into distress. Poor interface design, unreliable connectivity, privacy concerns, and cumbersome data entry constitute additional layers of workload that can lead to disengagement and negative psychological outcomes. For older adults and those with limited digital literacy, the effort demanded by technology operation may exceed the support it provides, exacerbating dependency, embarrassment, and loss of confidence (41, 42). This finding extends the concept of treatment burden to digital health: technical complexity is not merely a usability concern but an ethical issue that can undermine emotional safety and equity. When the demands of technology outweigh its benefits, patients experience not simply non-adoption but active psychological harm, including frustration, inferiority, and abandonment.

### 4.9 Clinical and policy implications

These findings carry direct implications for the design, evaluation, and implementation of cardiovascular digital interventions. First, emotional outcomes including reassurance, trust, loneliness, anxiety, and treatment burden should be routinely evaluated alongside clinical effectiveness and usability in trial frameworks and regulatory assessments (43). Second, system designs must ensure visible human oversight with clear, timely action pathways; patients need to know that their data are reviewed by responsive professionals who can intervene when necessary. Third, personalization must extend beyond demographic tailoring to encompass disease stage, health literacy, cultural context, and relational preferences. Fourth, technical reliability and low-friction design are ethical imperatives because complexity disproportionately burdens the most vulnerable users. Finally, digital tools should be integrated into existing clinical workflows rather than positioned as standalone replacements, preserving relational continuity and care coherence.

### 4.10 Strengths

This review has several strengths. Emotional support was treated as the primary phenomenon and was explicitly separated from enabling mechanisms (such as trust and perceived control) and negative outcomes (such as anxiety and burden). The combination of protocol-defined sensitizing concepts with inductively generated descriptive and analytical themes improved conceptual consistency without preventing unanticipated findings. PRISMA 2020 and ENTREQ reporting, duplicate screening and coding, retention of negative cases, and GRADE-CERQual judgments enhanced transparency. Finally, distinguishing the digital tool from the clinicians, peers, or family members connected through it prevented support mediated by technology from being misattributed to technology alone.

### 4.11 Limitations

Several limitations should be acknowledged. First, qualitative synthesis is interpretive and alternative theoretical lenses might produce different emphases. Second, reflexivity was poorly reported in many primary studies. Third, three studies examined anticipated rather than actual use. Fourth, only English- and Chinese-language studies were eligible. Fifth, grey literature was not searched, which may have increased publication bias, although backward reference searching was undertaken. Sixth, convenience samples, short interventions, and the heterogeneity of conditions and technologies limit condition-specific and long-term conclusions. Finally, because included reports varied in the detail of quotations and contextual description, the relative frequency or magnitude of emotional experiences cannot be inferred.

### 4.12 Future research directions

Future research should prioritize longitudinal qualitative designs that trace how emotional support experiences evolve as novelty effects wane and technical difficulties accumulate. Deeper investigation is needed into the emotional boundaries of conversational AI and immersive technologies, including their capacity to simulate empathy and their impact on relational continuity over extended periods. Implementation science frameworks should be applied to understand how organizational culture, clinician workload, reimbursement structures, and health system contexts shape the sociotechnical arrangements that produce emotional support. Additionally, patient-reported outcome measures specific to digital emotional support—encompassing reassurance, technology-mediated loneliness, perceived relational continuity, and digital treatment burden—require development and validation to enable quantitative assessment of these phenomena in clinical trials and quality improvement initiatives.

## 5 Conclusion

In conclusion, emotional support in cardiovascular digital health is not delivered by technology alone. It is co-produced when digital functions connect patients to credible oversight, understandable information, responsive relationships, and meaningful control. Conversely, unreliable, impersonal, opaque, or burdensome systems can amplify anxiety and disengagement. Digital interventions should therefore be designed and evaluated as sociotechnical systems in which emotional and relational outcomes are considered alongside clinical effectiveness and usability.

## Data Availability

The original contributions presented in the study are included in the article/Supplementary Material, further inquiries can be directed to the corresponding author/s.
