# Supplementary material for: How digital health tools shape emotional support experiences in cardiovascular care: a systematic review and thematic synthesis
Source: Front Cardiovasc Med. 2026 Aug 13;13:1923477. doi: 10.3389/fcvm.2026.1923477 (PMC13519404; doi:10.3389/fcvm.2026.1923477)
Supplement: Supplementary file 1 [file Datasheet1.docx]

***Supplementary Material***

[Appendix 1. Search strategies	3](#_Toc8537)

[Appendix 2. Methodological quality assessment of included studies	7](#_Toc27505)

[Appendix 3. CERQual summary of qualitative findings	8](#_Toc11342)

[Appendix 4. Completed PRISMA 2020 checklist	10](#_Toc15932)

[Appendix 5. Completed ENTREQ checklist	12](#_Toc13587)

# Appendix 1. Search strategies

| Databases | Terms |
| --- | --- |
| PubMed（458） | #1 "Digital Health"[MeSH] OR "Telemedicine"[MeSH] OR "Mobile Applications"[MeSH] OR "Wearable Electronic Devices"[MeSH] OR "Virtual Reality"[MeSH] OR "mHealth"[tiab] OR "mobile health"[tiab] OR "digital health tool*"[tiab] OR "telehealth"[tiab] OR "telemedicine"[tiab] OR "eHealth"[tiab] OR "smartphone app*"[tiab] OR "health app*"[tiab] OR "wearable device*"[tiab] OR "remote monitoring"[tiab] OR "virtual reality"[tiab] OR "immersive technology"[tiab]  #2 "Cardiovascular Diseases"[MeSH] OR "Heart Failure"[MeSH] OR "Myocardial Infarction"[MeSH] OR "Coronary Artery Disease"[MeSH] OR "Hypertension"[MeSH] OR "Arrhythmias, Cardiac"[MeSH] OR "cardiovascular disease*"[tiab] OR "heart disease*"[tiab] OR "cardiac disease*"[tiab] OR "heart failure"[tiab] OR "myocardial infarction"[tiab] OR "coronary heart disease"[tiab] OR "coronary artery disease"[tiab] OR "hypertension"[tiab] OR "cardiac rehabilitation"[tiab] OR "heart rehabilitation"[tiab]  #3 "Emotions"[MeSH] OR "Affect"[MeSH] OR "Psychological Support"[MeSH] OR "Social Support"[MeSH] OR "Empathy"[MeSH] OR "emotional support"[tiab] OR "emotional experience*"[tiab] OR "affective experience*"[tiab] OR "psychological support"[tiab] OR "psychosocial support"[tiab] OR "social support"[tiab] OR "peer support"[tiab] OR "empathy"[tiab] OR "compassion"[tiab] OR "sense of security"[tiab] OR "peace of mind"[tiab] OR "emotional well-being"[tiab] OR "loneliness"[tiab] OR "isolation"[tiab] OR "anxiety"[tiab] OR "depression"[tiab] OR "fear"[tiab] OR "distress"[tiab] OR "coping"[tiab] OR "resilience"[tiab] OR "motivation"[tiab] OR "self-efficacy"[tiab] OR "empowerment"[tiab] OR "trust"[tiab] OR "connectedness"[tiab] OR "belonging"[tiab]  #4 "Qualitative Research"[MeSH] OR "Narrative"[tiab] OR "experience"[tiab] OR "perception"[tiab] OR "perspective"[tiab] OR "attitude"[tiab] OR "feeling"[tiab] OR "lived experience"[tiab] OR "patient experience"[tiab] OR "user experience"[tiab]  #5 #1 AND #2 AND #3 AND #4 |
| Scopus（1115） | ( ("Digital Health") OR ("Telemedicine") OR ("Mobile Applications") OR ("Wearable Electronic Devices") OR ("Virtual Reality") OR ("mHealth") OR ("mobile health") OR ("digital health tool*") OR ("telehealth") OR ("telemedicine") OR ("eHealth") OR ("smartphone app*") OR ("health app*") OR ("wearable device*") OR ("remote monitoring") OR ("virtual reality") OR ("immersive technology") ) AND ( ("Cardiovascular Diseases") OR ("Heart Failure") OR ("Myocardial Infarction") OR ("Coronary Artery Disease") OR ("Hypertension") OR ("Arrhythmias, Cardiac") OR ("cardiovascular disease*") OR ("heart disease*") OR ("cardiac disease*") OR ("heart failure") OR ("myocardial infarction") OR ("coronary heart disease") OR ("coronary artery disease") OR ("hypertension") OR ("cardiac rehabilitation") OR ("heart rehabilitation") ) AND ( ("emotional support") OR ("emotional experience*") OR ("affective experience*") OR ("psychological support") OR ("psychosocial support") OR ("social support") OR ("peer support") OR ("empathy") OR ("compassion") OR ("sense of security") OR ("peace of mind") OR ("emotional well-being") OR ("loneliness") OR ("isolation") OR ("anxiety") OR ("depression") OR ("fear") OR ("distress") OR ("coping") OR ("resilience") OR ("motivation") OR ("self-efficacy") OR ("empowerment") OR ("trust") OR ("connectedness") OR ("belonging") ) AND ( ("qualitative" W/3 ("research" OR "study" OR "interview")) OR ("Narrative") OR ("experience") OR ("perception") OR ("perspective") OR ("attitude") OR ("feeling") OR ("lived experience") OR ("patient experience") OR ("user experience") ) |
| Embase（532） | #1 ('digital health'/mj OR 'telemedicine'/mj OR 'mobile application'/mj OR 'wearable electronic device'/mj OR 'virtual reality'/mj OR 'mHealth':ti,ab OR 'mobile health':ti,ab OR 'digital health tool*':ti,ab OR telehealth:ti,ab OR telemedicine:ti,ab OR eHealth:ti,ab OR 'smartphone app*':ti,ab OR 'health app*':ti,ab OR 'wearable device*':ti,ab OR 'remote monitoring':ti,ab OR 'virtual reality':ti,ab OR 'immersive technology':ti,ab)  #2 ('cardiovascular disease'/mj OR 'heart failure'/mj OR 'myocardial infarction'/mj OR 'coronary artery disease'/mj OR hypertension/mj OR 'heart arrhythmia'/mj OR 'cardiovascular disease*':ti,ab OR 'heart disease*':ti,ab OR 'cardiac disease*':ti,ab OR 'heart failure':ti,ab OR 'myocardial infarction':ti,ab OR 'coronary heart disease':ti,ab OR 'coronary artery disease':ti,ab OR hypertension:ti,ab OR 'cardiac rehabilitation':ti,ab OR 'heart rehabilitation':ti,ab)  #3 ('emotion'/mj OR 'affect'/mj OR 'psychological support'/mj OR 'social support'/mj OR 'empathy'/mj OR 'emotional support':ti,ab OR 'emotional experience*':ti,ab OR 'affective experience*':ti,ab OR 'psychological support':ti,ab OR 'psychosocial support':ti,ab OR 'social support':ti,ab OR 'peer support':ti,ab OR empathy:ti,ab OR compassion:ti,ab OR 'sense of security':ti,ab OR 'peace of mind':ti,ab OR 'emotional well-being':ti,ab OR loneliness:ti,ab OR isolation:ti,ab OR anxiety:ti,ab OR depression:ti,ab OR fear:ti,ab OR distress:ti,ab OR coping:ti,ab OR resilience:ti,ab OR motivation:ti,ab OR 'self-efficacy':ti,ab OR empowerment:ti,ab OR trust:ti,ab OR connectedness:ti,ab OR belonging:ti,ab)  #4 ('qualitative research'/mj OR 'narrative':ti,ab OR experience:ti,ab OR perception:ti,ab OR perspective:ti,ab OR attitude:ti,ab OR feeling:ti,ab OR 'lived experience':ti,ab OR 'patient experience':ti,ab OR 'user experience':ti,ab)  #5 #1 AND #2 AND #3 AND #4 |
| Web of Science  （1491） | TS = (("digital health" OR "digital medicine" OR "telehealth" OR "telemedicine" OR "mHealth" OR "mobile health" OR "eHealth" OR "mobile application*" OR "smartphone app*" OR "health app*" OR "wearable device*" OR "wearable technology" OR "remote monitoring" OR "remote patient monitoring" OR "virtual reality" OR "immersive virtual reality" OR "VR" OR "augmented reality" OR "digital intervention*" OR "digital tool*" OR "digital platform*" OR "telecare" OR "telerehabilitation" OR "digital therapeutics") AND ("cardiovascular disease*" OR "heart disease*" OR "cardiac disease*" OR "heart failure" OR "myocardial infarction" OR "coronary heart disease" OR "coronary artery disease" OR "hypertension" OR "cardiac arrhythmia*" OR "atrial fibrillation" OR "cardiac rehabilitation" OR "heart rehabilitation" OR "cardiac telerehabilitation" OR "coronary disease*" OR "ischemic heart disease" OR "cerebrovascular disease*" OR "stroke" OR "transient ischemic attack") AND ("emotional support" OR "emotional experience*" OR "affective experience*" OR "psychological support" OR "psychosocial support" OR "social support" OR "peer support" OR "empathy" OR "compassion" OR "sense of security" OR "peace of mind" OR "emotional well-being" OR "loneliness" OR "isolation" OR "anxiety" OR "depression" OR "fear" OR "distress" OR "coping" OR "resilience" OR "motivation" OR "self-efficacy" OR "empowerment" OR "trust" OR "connectedness" OR "belonging" OR "emotional benefit*" OR "emotional need*" OR "emotional impact*" OR "emotional response*" OR "emotional engagement" OR "therapeutic alliance" OR "patient-provider relationship" OR "human connection") AND ("qualitative" OR "interview*" OR "focus group*" OR "narrative" OR "phenomenolog*" OR "grounded theory" OR "content analysis" OR "thematic analysis" OR "experience" OR "perception*" OR "perspective*" OR "attitude*" OR "feeling*" OR "lived experience" OR "patient experience" OR "user experience")) |
| China National Knowledge Infrastructure（20） | TKA=数字健康 + 数字医学 + 远程健康 + 远程医疗 + 移动健康 + 手机健康 + 电子健康 + 移动应用 + 智能手机应用 + 健康应用 + 可穿戴设备 + 可穿戴技术 + 远程监测 + 远程患者监测 + 虚拟现实 + 沉浸式虚拟现实 + VR + 增强现实 + 数字干预 + 数字工具 + 数字平台 + 远程护理 + 远程康复 + 数字治疗学 AND TKA=心血管疾病 + 心脏病 + 心脏疾病 + 心力衰竭 + 心肌梗死 + 冠心病 + 冠状动脉疾病 + 高血压 + 心律失常 + 房颤 + 心脏康复 + 心脏复健 + 心脏远程康复 + 冠状动脉疾病 + 缺血性心脏病 + 脑血管疾病 + 中风 + 短暂性脑缺血发作 AND TKA=情感支持 + 情感体验 + 情绪体验 + 心理支持 + 心理社会支持 + 社会支持 + 同伴支持 + 共情 + 同情 + 安全感 + 心理平静 + 情绪健康 + 孤独 + 孤立 + 焦虑 + 抑郁 + 恐惧 + 痛苦 + 应对 + 韧性 + 动机 + 自我效能 + 赋权 + 信任 + 联系感 + 归属感 + 情感益处 + 情感需求 + 情感影响 + 情感反应 + 情感参与 + 治疗联盟 + 医患关系 + 人际联系 AND TKA=定性 + 访谈 + 焦点小组 + 叙事 + 现象学 + 扎根理论 + 内容分析 + 主题分析 + 体验 + 感知 + 观点 + 态度 + 感受 + 亲身经历 + 患者体验 + 用户体验 |
| Wanfang Database  （75） | 数字健康 or 数字医学 or 远程健康 or 远程医疗 or 移动健康 or 手机健康 or 电子健康 or 移动应用 or 智能手机应用 or 健康应用 or 可穿戴设备 or 可穿戴技术 or 远程监测 or 远程患者监测 or 虚拟现实 or 沉浸式虚拟现实 or VR or 增强现实 or 数字干预 or 数字工具 or 数字平台 or 远程护理 or 远程康复 or 数字治疗学 and 心血管疾病 or 心脏病 or 心脏疾病 or 心力衰竭 or 心肌梗死 or 冠心病 or 冠状动脉疾病 or 高血压 or 心律失常 or 房颤 or 心脏康复 or 心脏复健 or 心脏远程康复 or 冠状动脉疾病 or 缺血性心脏病 or 脑血管疾病 or 中风 or 短暂性脑缺血发作 and 情感支持 or 情感体验 or 情绪体验 or 心理支持 or 心理社会支持 or 社会支持 or 同伴支持 or 共情 or 同情 or 安全感 or 心理平静 or 情绪健康 or 孤独 or 孤立 or 焦虑 or 抑郁 or 恐惧 or 痛苦 or 应对 or 韧性 or 动机 or 自我效能 or 赋权 or 信任 or 联系感 or 归属感 or 情感益处 or 情感需求 or 情感影响 or 情感反应 or 情感参与 or 治疗联盟 or 医患关系 or 人际联系 and 定性 or 访谈 or 焦点小组 or 叙事 or 现象学 or 扎根理论 or 内容分析 or 主题分析 or 体验 or 感知 or 观点 or 态度 or 感受 or 亲身经历 or 患者体验 or 用户体验 |
| Weipu  Database  (101） | TKA=数字健康 + 数字医学 + 远程健康 + 远程医疗 + 移动健康 + 手机健康 + 电子健康 + 移动应用 + 智能手机应用 + 健康应用 + 可穿戴设备 + 可穿戴技术 + 远程监测 + 远程患者监测 + 虚拟现实 + 沉浸式虚拟现实 + VR + 增强现实 + 数字干预 + 数字工具 + 数字平台 + 远程护理 + 远程康复 + 数字治疗学 AND TKA=心血管疾病 + 心脏病 + 心脏疾病 + 心力衰竭 + 心肌梗死 + 冠心病 + 冠状动脉疾病 + 高血压 + 心律失常 + 房颤 + 心脏康复 + 心脏复健 + 心脏远程康复 + 冠状动脉疾病 + 缺血性心脏病 + 脑血管疾病 + 中风 + 短暂性脑缺血发作 AND TKA=情感支持 + 情感体验 + 情绪体验 + 心理支持 + 心理社会支持 + 社会支持 + 同伴支持 + 共情 + 同情 + 安全感 + 心理平静 + 情绪健康 + 孤独 + 孤立 + 焦虑 + 抑郁 + 恐惧 + 痛苦 + 应对 + 韧性 + 动机 + 自我效能 + 赋权 + 信任 + 联系感 + 归属感 + 情感益处 + 情感需求 + 情感影响 + 情感反应 + 情感参与 + 治疗联盟 + 医患关系 + 人际联系 AND TKA=定性 + 访谈 + 焦点小组 + 叙事 + 现象学 + 扎根理论 + 内容分析 + 主题分析 + 体验 + 感知 + 观点 + 态度 + 感受 + 亲身经历 + 患者体验 + 用户体验 |

# Appendix 2. Methodological quality assessment of included studies

**Supplementary Table 2.** Methodological quality assessment of included studies

| Included study | Q1 | Q2 | Q3 | Q4 | Q5 | Q6 | Q7 | Q8 | Q9 | Q10 |
| --- | --- | --- | --- | --- | --- | --- | --- | --- | --- | --- |
| Blomqvist et al.2025 | Y | Y | Y | Y | Y | Y | Y | Y | Y | Y |
| Cher et al. 2020 | Y | Y | Y | Y | U | U | U | Y | Y | Y |
| Choo et al. 2025 | Y | Y | Y | Y | U | Y | U | Y | Y | Y |
| Chu et al. 2024 | Y | Y | Y | Y | U | U | U | Y | Y | Y |
| Foreman et al. 2025 | Y | Y | Y | Y | U | U | U | Y | Y | Y |
| Lan et al. 2021 | Y | Y | Y | Y | U | U | U | Y | Y | Y |
| Lao and Chair. 2022 | Y | Y | Y | Y | U | U | U | Y | Y | Y |
| Madujibeya et al. 2023 | Y | Y | Y | Y | U | U | U | Y | Y | Y |
| Micheluzzi et al. 2025 | Y | Y | Y | Y | Y | U | U | Y | Y | Y |
| Neumann et al. 2024 | Y | Y | Y | Y | Y | U | U | Y | Y | Y |
| Nilsson et al. 2023 | Y | Y | Y | Y | U | U | U | Y | Y | Y |
| O‘Shea et al. 2020 | Y | Y | Y | Y | U | U | U | Y | Y | Y |
| Schmaderer et al. 2021 | Y | Y | Y | Y | U | U | U | Y | Y | Y |
| Sivakumar et al. 2023 | Y | Y | Y | Y | U | U | U | Y | Y | Y |
| Son et al. 2020 | Y | Y | Y | Y | U | U | U | Y | Y | Y |
| Su et al. 2023 | Y | Y | Y | Y | Y | U | U | Y | Y | Y |
| Tang et al. 2022 | Y | Y | Y | Y | U | U | U | Y | Y | Y |
| Trivedi et al.2025 | Y | Y | Y | Y | Y | U | U | Y | Y | Y |
| Number of Y | 18 | 18 | 18 | 18 | 5 | 2 | 1 | 18 | 18 | 18 |
| Percentage (%) | 100 | 100 | 100 | 100 | 28 | 11 | 6 | 100 | 100 | 100 |

JBI checklist items: Q1 = Congruity between philosophical perspective and research question; Q2 = Congruity between research methodology and research question; Q3 = Congruity between data collection methods and research question; Q4 = Congruity between data representation and analysis; Q5 = Statement of researcher’s positioning and reflexivity; Q6 = Statement of researcher’s cultural/theoretical positioning; Q7 = Adequate description of the relationship between researcher and participants; Q8 = Adequate representation of participants and their voices; Q9 = Reporting of ethical approval and procedures; Q10 = Conclusions derived from data analysis.

# Appendix 3. CERQual summary of qualitative findings

**Supplementary Table 3.** CERQual summary of qualitative findings

| **Synthesised finding** | **CERQual confidence** | **Contributing studies** | **Main concerns** | **Explanation of judgement** |
| --- | --- | --- | --- | --- |
| 1. Monitoring translates into reassurance when linked to visible professional oversight | High | Telerehabilitation [13], RPM [7], HF apps [10,11], AF monitoring [8], cardiac rehab [1,14] | Methodological limitations: some studies used convenience sampling or recruited engaged users only | Consistent evidence across different conditions (HF, CAD, AF, hypertension) and technologies. Findings converge on the mechanism that visible responsiveness, not monitoring per se, generates reassurance. Negative cases (e.g., distrust of devices [7]) reinforce rather than undermine the finding. Data are sufficient and directly relevant. |
| 2. Understandable information and feedback restore agency and emotional balance | High | HF apps [5,12], hypertension app [17], lifestyle app [3], CHD eHealth [4], AF tools [8], general HF needs [9] | Methodological limitations: three studies assessed anticipated rather than actual use [9,12,16] | Strong convergence across diverse populations. The finding that actionable, personalised information enhances perceived control and reduces distress is consistently reported. Negative cases (information overload, generic content) provide clear boundary conditions [8,12,18]. Data are rich and directly relevant to cardiovascular self-management. |
| 3. Digital connections provide support only when human relationships remain humane and responsive | Moderate to High | Clinician support [4,7,11,13,15,17]; peer support [4,9]; family support [12,15,17,18]; AI limitations [8]; cultural safety [2] | Methodological limitations: reflexivity poorly reported; peer- and family-support evidence less extensive than clinician-support evidence | Highly coherent across studies: patients consistently value human responsiveness over autonomous digital functions. Clinician-support evidence is robust (rated High). However, evidence for peer- and family-mediated support is narrower in scope, and findings on AI and cultural safety come from fewer studies. Downgraded for adequacy (peer/family domains) and relevance (AI findings are preliminary). |
| 4. Personalisation, enjoyment, and encouragement sustain motivation and hope | Moderate | VR [14], physical-activity apps [6,13], tailored feedback [1,3,4], rehabilitation programmes [13,14] | Methodological limitations: most studies had short intervention periods (≤3–6 months); longer-term durability of motivational effects is uncertain | Consistent evidence that enjoyment, visible progress, and positive encouragement support intrinsic motivation and hope. The mechanism is plausible and aligns with self-determination theory. However, evidence comes from fewer studies compared to other themes, and most assessed short-term use. Downgraded for adequacy (limited number of studies specifically addressing positive emotional outcomes) and relevance (long-term sustainability not tested). |
| 5. Technical burden, information overload, privacy concerns, and distrust can reverse support into anxiety and disengagement | High | Technical burden [1,6,8–10,14,18]; information overload [8,12,18]; privacy/cost concerns [9,12,16]; distrust [2,7]; discontinuation studies [18] | Methodological limitations: some studies did not include perspectives of non-users or those who completely dropped out (except Lan et al. [18]) | Very strong and consistent evidence across all technology types (apps, RPM, VR, AI) and populations. The "reverse pathway" — from intended support to frustration, anxiety, and abandonment — is explicitly described in multiple studies. Data are sufficient, and the finding is highly relevant to implementation. The inclusion of a discontinuation-specific study [18] strengthens the finding. |

Note: CERQual (Confidence in the Evidence from Reviews of Qualitative research) assessments are based on four components: methodological limitations, coherence, adequacy of data, and relevance. Confidence ratings range from High to Very Low

# Appendix 4. Completed PRISMA 2020 checklist

| Item | PRISMA topic | How addressed | Location |
| --- | --- | --- | --- |
| 1 | Title | Identifies the report as a systematic review and thematic synthesis. | Title |
| 2 | Abstract | Structured summary includes background, objective, sources, eligibility, synthesis, results, and implications. | Abstract |
| 3 | Rationale | Explains why emotional support requires synthesis beyond usability and barrier–facilitator reviews. | Introduction |
| 4 | Objectives | States three review questions concerning experiences, mechanisms, and contextual conditions. | Introduction |
| 5 | Eligibility criteria | PICo inclusion and exclusion criteria are reported in a dedicated table. | Methods, Table 1 |
| 6 | Information sources | Seven bibliographic databases and backward reference checking are described; final search date is reported. | Methods; Appendix 1 |
| 7 | Search strategy | Full database-specific search strategies and yields are provided. | Appendix 1 |
| 8 | Selection process | Independent duplicate title/abstract and full-text screening are described. | Methods |
| 9 | Data collection process | Standardized extraction of study, tool, provider, context, and qualitative findings is described. | Methods |
| 10a-b | Data items | Outcomes and contextual variables, including human support provider, are specified. | Methods; Table 2 |
| 11 | Risk of bias assessment | JBI qualitative checklist and use of appraisal in interpretation are described. | Methods; Appendix 2 |
| 13a-f | Synthesis methods | Thomas and Harden thematic synthesis, deductive–inductive coding, negative cases, and comparison across contexts are described. | Methods |
| 15 | Certainty assessment | GRADE-CERQual domains and judgments are described. | Methods; Table 3; Appendix 3 |
| 16a-b | Study selection | Record counts, screening stages, exclusions, and flow diagram are reported. | Results; Figure 1 |
| 17 | Study characteristics | Countries/regions, populations, technologies, contexts, and support providers are reported. | Results; Table 2 |
| 18 | Risk of bias in studies | JBI findings and reflexivity limitations are summarized. | Results; Appendix 2 |
| 19-20 | Results of syntheses | Five analytical themes, similarities/differences, moderators, and CERQual judgments are reported. | Results; Table 3 |
| 23a-d | Discussion | Findings are interpreted against previous reviews; strengths, limitations, implications, and future research are reported. | Discussion |
| 24a-c | Registration and protocol | PROSPERO registration number is reported. | Methods |
| 25 | Support | No funding is declared. | Title page |
| 26 | Competing interests | No competing interests are reported in the manuscript declarations. | Declarations |
| 27 | Availability of data/materials | Supplementary strategies, appraisal, CERQual, and reporting checklists are supplied. | Supplementary Material |

# Appendix 5. Completed ENTREQ checklist

| Item | ENTREQ domain | How addressed | Location |
| --- | --- | --- | --- |
| 1 | Aim | Review aim and questions are explicitly stated. | Abstract; Introduction |
| 2 | Synthesis methodology | Systematic review with Thomas and Harden thematic synthesis. | Methods |
| 3 | Approach to searching | Comprehensive database searching plus backward reference checking; grey literature exclusion justified. | Methods |
| 4 | Inclusion criteria | PICo table distinguishes population, core phenomenon, mechanisms, negative boundaries, context, and study type. | Methods; Table 1 |
| 5 | Data sources | Seven databases searched from inception to December 2025. | Methods; Appendix 1 |
| 6 | Electronic search strategy | Full strategies and record yields are reported for each database. | Appendix 1 |
| 7 | Study screening methods | Two-stage independent screening by two reviewers. | Methods |
| 8 | Study characteristics | Study, population, tool, exposure, provider, and key finding reported. | Table 2 |
| 9 | Study selection results | PRISMA flow and numerical selection results reported. | Results; Figure 1 |
| 10 | Rationale for appraisal | Quality used to inform interpretation and CERQual rather than automatic exclusion. | Methods |
| 11 | Appraisal items | JBI 10-item checklist. | Methods; Appendix 2 |
| 12 | Appraisal process | Two-reviewer appraisal and interpretation described. | Methods |
| 13 | Appraisal results | Overall quality and reflexivity concerns reported. | Results; Appendix 2 |
| 14 | Data extraction | Standardized extraction including first- and second-order qualitative findings and human support provider. | Methods |
| 15 | Software | Not used as an analytic determinant; synthesis conducted through reviewer coding and comparison. | Methods |
| 16 | Number of reviewers | Two reviewers coded findings; disagreements resolved through discussion. | Methods |
| 17 | Coding | Line-by-line deductive–inductive coding with sensitizing concepts and new codes. | Methods |
| 18 | Study comparison | Codes and descriptive categories compared across technologies, conditions, and positive/negative cases. | Methods; Results |
| 19 | Derivation of themes | Descriptive categories were inductive; analytical themes explain interactions among tools, people, and context. | Methods |
| 20 | Quotations | Primary-study interpretations and participant quotations were eligible data; manuscript reports study-linked synthesis. | Methods; Results |
| 21 | Synthesis output | Five analytical themes, six moderators, CERQual summary, and integrative conceptual model. | Results; Figure 2 |

**References**

# [1]O'Shea O, Woods C, McDermott L, et al. A qualitative exploration of cardiovascular disease patients' views and experiences with an eHealth cardiac rehabilitation intervention: The PATHway Project. PLoS One. 2020;15(7):e0235274. Published 2020 Jul 6. doi:10.1371/journal.pone.0235274

# [2]Foreman MA, Ross A, Burgess APH, Myneni S, Franklin A. Barriers and Facilitators of Digital Health Use for Self-Management of Hypertensive Disorders by Black Pregnant Women. AMIA Annu Symp Proc. 2025;2024:433-442. Published 2025 May 22.

# [3]Choo J, Noh S, Shin Y. Evaluating Feasibility and Acceptability of the "My HeartHELP" Mobile App for Promoting Heart-Healthy Lifestyle Behaviors: Mixed Methods Study. JMIR Form Res. 2025;9:e66108. Published 2025 May 2. doi:10.2196/66108

# [4]Su JJ, Paguio J, Baratedi WM, Abu-Odah H, Batalik L. Experience of coronary heart disease patients with a nurse-led eHealth cardiac rehabilitation: Qualitative process evaluation of a randomized controlled trial. Heart Lung. 2023;57:214-221. doi:10.1016/j.hrtlng.2022.10.005

# [5]Schmaderer M, Miller JN, Mollard E. Experiences of Using a Self-management Mobile App Among Individuals With Heart Failure: Qualitative Study. JMIR Nurs. 2021;4(3):e28139. Published 2021 Aug 9. doi:10.2196/28139

# [6]Blomqvist A, Strömberg A, Lundberg M, Bäck M, Jaarsma T, Klompstra L. Exploring user experience: A qualitative analysis of the use of a physical activity support app for people with heart failure. PLoS One. 2025;20(5):e0309577. Published 2025 May 22. doi:10.1371/journal.pone.0309577

# [7]Chu F, Stark A, Telzak A, Rikin S. Patient Experience in a Remote Patient Monitoring Program for Hypertension: A Qualitative Study. Am J Hypertens. 2024;37(11):861-867. doi:10.1093/ajh/hpae086

# [8]Trivedi R, Shaw T, Sheahen B, Chow CK, Laranjo L. Patient Perspectives on Conversational Artificial Intelligence for Atrial Fibrillation Self-Management: Qualitative Analysis. J Med Internet Res. 2025;27:e64325. Published 2025 Mar 12. doi:10.2196/64325

# [9]Sivakumar B, Lemonde M, Stein M, Mak S, Al-Hesayen A, Arcand J. Patient perspectives on the use of mobile apps to support heart failure management: A qualitative descriptive study. PLoS One. 2023;18(5):e0285659. Published 2023 May 11. doi:10.1371/journal.pone.0285659

# [10]Neumann A, Steiner B, Verket M, et al. Patients' expectations and experiences with the usage of a self-care application for heart failure: A qualitative interview study. Digit Health. 2024;10:20552076241299649. Published 2024 Nov 20. doi:10.1177/20552076241299649

# [11]Patients’ Experiences Using a Mobile Health App for Self-Care of Heart Failure in a Real-World Setting: Qualitative Analysis

# [12]Patients’ needs and perspectives for using mobile phone interventions to improve heart failure self-care: A qualitative study

# [13]Nilsson U, Öberg B, Bäck M. Patients' Perceptions of Exercise-Based Cardiac Telerehabilitation after a Myocardial Infarction-A Qualitative Study. Int J Environ Res Public Health. 2023;20(7):5420. Published 2023 Apr 6. doi:10.3390/ijerph20075420

# [14]Micheluzzi V, Casu G, Burrai F, et al. The experience of immersive virtual reality in patients with heart failure during cardiac rehabilitation: a qualitative study. Front Med (Lausanne). 2025;12:1578399. Published 2025 May 14. doi:10.3389/fmed.2025.1578399

# [15]Lao SSW, Chair SY. The feasibility of smartphone-based application on cardiac rehabilitation for Chinese patients with percutaneous coronary intervention in Macau: a qualitative evaluation. Int J Qual Stud Health Well-being. 2022;17(1):2023940. doi:10.1080/17482631.2021.2023940

# [16]Cher BP, Kembhavi G, Toh KY, et al. Understanding the Attitudes of Clinicians and Patients Toward a Self-Management eHealth Tool for Atrial Fibrillation: Qualitative Study. JMIR Hum Factors. 2020;7(3):e15492. Published 2020 Sep 17. doi:10.2196/15492

# [17]Tang C, Zhang X F, Li B X, et al. A qualitative study on the application experience of mobile health platforms in hypertensive patients[J]. Contemporary Nurse, 2022, 29(10): 61-66. DOI:10.19791/j.cnki.1006-6411.2022.10.018.

# [18]Lan CH, Qiu XQ, Shen M, et al. A qualitative study on the real experience of patients with low willingness to accept mobile health apps for heart failure[J]. Chinese Journal of New Clinical Medicine, 2021, 14(12): 1238-1241.
